# A Curriculum to Teach Resilience Skills to Medical Students During Clinical Training

**DOI:** 10.15766/mep_2374-8265.10975

**Published:** 2020-09-30

**Authors:** Amber Bird, Oana Tomescu, Sonia Oyola, Jennifer Houpy, Irsk Anderson, Amber Pincavage

**Affiliations:** 1 Assistant Professor of Medicine, Department of Medicine, Perelman School of Medicine at the University of Pennsylvania; 2 Associate Professor of Medicine, Department of Medicine, Perelman School of Medicine at the University of Pennsylvania; 3 Assistant Professor, Department of Family Medicine, University of Chicago Pritzker School of Medicine; 4 Internal Medicine Resident, Department of Medicine, University of Chicago Pritzker School of Medicine; 5 Assistant Professor, Department of Medicine, University of Chicago Pritzker School of Medicine; 6 Associate Professor, Department of Medicine, University of Chicago Pritzker School of Medicine

**Keywords:** Wellness, Clerkship, Clinical Clerkship, Burnout, Resilience, Microblogging, Reflection/Narrative Medicine, Well-Being/Mental Health

## Abstract

**Introduction:**

Burnout in medical students is extensive and a critical issue. It is associated with increased rates of depression, suicide, and poor perception of the educational environment. Enhancing resilience, the ability to adapt well in the face of adversity, is a potential tool to mitigate burnout and improve medical student wellness.

**Methods:**

Our resilience curriculum consisted of facilitated workshops to cultivate resilience in medical students during their core clerkship rotations. This curriculum served as an introduction to the concept of resilience and taught skills to cultivate resilience and promote wellness. The sessions allowed for identification of and reflection on stressors in the clinical learning environment, including straining team dynamics, disappointment, and uncertainty. Educational sessions included resilience skill-building exercises for managing expectations, letting go of negative emotions, dealing with setbacks, and finding meaning in daily work. Associated materials included lesson plans for small-group facilitators, learner pre- and postcurriculum surveys, and a social media activity guide.

**Results:**

This curriculum was delivered to 144 clerkship students at two academic institutions over the 2017–2018 academic year. Sessions were well received by medical students, with the majority of students stating that the sessions should continue. The majority of attendees found the sessions valuable and learned new ways to approach challenges.

**Discussion:**

Students valued connecting with peers and feeling less alone through their participation. A challenge was constructing a setting conducive to comfortable reflection for all learners. Not all students found these sessions necessary. Sessions may have improved resilience levels.

## Educational Objectives

By the end of this activity, learners will be able to:

1. Define resilience and identify how it applies to adversities encountered during clinical training for medical students.
2. Demonstrate focused skill-building exercises to promote resilience behaviors.
3. Reflect on adversities, stressful situations, and finding meaning in clerkship rotations.
4. Define a goal and an expectation and identify the difference between them.
5. Identify how to deconstruct goals into realistic, measurable portions and how to consciously set expectations.
6. Identify healthy coping strategies after challenging team dynamics.
7. Practice compassionate listening when discussing setbacks and disappointments.
8. Identify ways to find meaning in clerkship work through practice of reflection and gratitude.
9. Describe some key health and mental health benefits of providing compassionate care for patients and health care providers.

## Introduction

Burnout and depression are prevalent among medical students. Burnout in students has been correlated with depression and a less positive perception of the learning environment.^1^ Depressive symptoms have been associated with stress from the medical school environment more than personal factors. Although stress, burnout, and depression in medical students have been well described, there is more uncertainty about medical student resilience.^1,2^ Resilience is the ability to cope well with adversity and can be learned.^3^ In one study, resilience in third- and fourth-year medical students was lower than in the general population and higher in students reporting no symptoms of burnout.^1^ Fostering resilience therefore has been advocated to help mitigate stressors, burnout, and difficult experiences. While elective educational experiences in resilience have been described in the medical literature, to date no curricula have explored a skills-based approach to resilience education during clerkship years for medical students.^4^ Resilience skills can be particularly beneficial for clinical experiences in medical school as medical students encounter new stressors and become at risk for burnout.

Our curriculum to help cultivate resilience in students consisted of a series of skill-based workshops and exercises. The curriculum taught the following skills: managing expectations, coping with difficult team interactions, finding meaning in daily work, and dealing with disappointment and the unexpected. Sessions combine reflection, mindfulness, didactics, and peer discussion. The workshop series consisted of four individual small-group sessions. Each session took approximately 60 minutes, with a 10-minute introduction and review of the prior workshop, a 15-minute skill-building lesson, a 10-minute practical application, a 15-minute small-group discussion and brainstorming, and a 5-minute wrap-up. The sessions were designed for small groups of 10–20 participants. We also included a reflection exercise on Yammer, a microblogging platform compliant with the Health Insurance Portability and Accountability Act, or on Canvas. Students practiced positive psychology by posting a positive patient advocacy moment with faculty moderation.^5,6^

The curriculum was developed based on precurriculum survey responses from clerkship students at two institutions assessing resilience levels and perceived stressors in the clinical environment.^7^ The purpose of this survey was to assess student perception of clinical events and experiences during medical training that contributed to burnout. At both universities, more than 80% of clerkship students reported experiencing stress related to difficult team and patient interactions, perceived failure on clinical rotations, and lack of meaning or purpose in their clerkship work. The cases used in the skill-building exercises were based on stressful clinical events commonly reported by trainees, as well as on our personal experience educating and mentoring students and developing a resident curriculum focused on resilience skills.^8^ The small-group sessions and skill-building exercises were developed on the basis of existing literature on resilience and positive psychology, review of a resilience curriculum developed for internal medicine residents as adapted from undergraduate educational material first created by Dr. Alex Lickerman, and stressful aspects of our clerkship training as identified by clerkship students.^8–19^ All content areas incorporated core virtues of positive psychology, including intellectual strengths, interpersonal strengths, and temperance strengths. Positive psychology research has suggested that a team-based approach led by a resilience coach results in a more sustained improvement in well-being.^9,10^ For this reason, one or two facilitators familiar with the fields of positive psychology and resilience led each small-group session. Facilitators at both institutions had attended at least one faculty development session on mindfulness or resilience techniques through their institution; however, no facilitators had professional training in psychology or mental health counseling.

This workshop series can be implemented as an independent curriculum or as an addition to an established wellness curriculum for medical students. The facilitator role is intended for a faculty member directly involved in medical student education, particularly those familiar with clerkship training requirements.

## Methods

The target audience for this curriculum included beginner, intermediate, and advanced learners. Prior exposure to clinical medicine and issues of team-based care, direct communication, and care for a patient were recommended for the workshop sessions but were not mandatory.

Prior to initiating this workshop series, we identified medical school support by securing protected space for small-group sessions and discussing existing frameworks for referral to medical school leadership and student counseling resources for students identified as having high levels of burnout or distress.

Given the personal nature of these workshops, we delivered educational content in small-group settings. We secured a space separate from the standard clinical setting and reminded students to keep information shared during sessions confidential. We used a pair-and-share method to engage participants during reflection on sensitive topics.

We delivered this workshop series during dedicated lecture time in the core clerkship rotations for medical students. All sessions consisted of 10–20 clerkship students and took place in a small lecture hall or classroom. Each session was allotted 60 minutes: a 10-minute introduction, a 40-minute small-group skill-building exercise, and a 10-minute wrap-up. Participation in workshop sessions by clerkship students was voluntary at both institutions.

Prior to the first workshop, we prepared copies of the Connor-Davidson Resilience Scale (CD-RISC; optional) to measure and trend trainee resilience scores.^20^ We ensured all audiovisual equipment in the room had projection capabilities prior to the session. Prior to conducting the small-group sessions, we reviewed the instructions for facilitators. At the start of the first small-group session, we distributed a precurriculum survey to learners to assess their engagement and past experience with resilience training and stressful events during clinical training. During the four workshop sessions, we used skill-building exercise worksheets to introduce learners to the intended resilience topic and skill-building exercise for each workshop. At the conclusion of the final workshop, we distributed a workshop evaluation.

The first session defined resilience and the practice of setting realistic expectations. The second session addressed challenging team dynamics, while the third session focused on identification, processing, and moving on following disappointments and setbacks. The fourth session focused on finding meaning in daily work. These session topics were chosen based on our experience with resilience skill-building areas applicable to medical student clerkship training and existing literature including our needs assessment data.^7^ The sessions could be delivered in a variety of orders but always had to begin with the introduction to the concept of resilience.

• Distribute CD-RISC (Appendix A) to participants before starting the first small-group session (optional step).^19^ Provide background on the creation and validation of the scoring tool.
  ○ If allowing trainees to score their own scales, provide handout with scoring instructions.
  ○ After scoring is complete, provide general information on the variety of resilience scores.
  ○ Complete this step at the beginning of the first small-group session; it is an optional component for use if trending of scores is desired.
• Session 1: Introduction to Resilience, Setting Realistic Expectations.
  ○ Provide all students with the curriculum presurvey (Appendix B).
  ○ Provide an introduction to the concept of resilience.
  ○ Destigmatize the concept of resilience and stress and that system approaches to wellness are extremely important. Challenges will always exist within the health care system, but these skills allow students to tackle those challenges.
  ○ Define and give examples of resilience and how it plays a role in student well-being.
  ○ Introduce the first skill-building exercise, creating realistic expectations (Appendix C).
  ○ Ask participants to brainstorm areas during clerkship training where their expectations did not align with the reality of their experience.
  ○ Have small-groups discuss the impact of expectations on clinical rotations. Practice examples of setting realistic expectations.
• Session 2: Difficult Team Interactions.
  ○ Review concepts discussed in session 1. Allow participants to share their personal experience in applying skills learned from session 1 during the time between workshop sessions.
  ○ Provide background on difficult team interactions and impact on students during clerkship years.
  ○ Ask participants to brainstorm categories of stressful events experienced during clerkship training. Have participants share these categories with the group.
  ○ Use the session 2 lesson plan (Appendix D) to provide participants with the potential impact of these events on personal, patient, and system well-being.
  ○ Provide participants with the difficult team interactions pocket card (Appendix E). It is helpful to reserve time at the end of this session to discuss avenues available for reporting inappropriate, discriminatory, or abusive behavior encountered during clinical training.
• Session 3: Dealing With Disappointments and Setbacks.
  ○ Use the lesson plan for disappointments and setbacks (Appendix F) to review concepts of compassion and the benefits of compassionate care for both patients and health care providers.
  ○ Review the types of techniques and exercises, including loving-kindness meditation, Tonglen attitude/breathing, journaling, reflective writing, and compassionate listening, that can promote and enhance compassion and assist students in managing a disappointment and/or setback.
  ○ Review and practice compassionate listening (Appendix G) by pairing the students and giving them the opportunity to listen to each other's challenging clinical experience in a private setting.
  ○ End the session with a large-group debrief, where students are asked about their experience with compassionate listening.
• Session 4: Finding Meaning.
  ○ Review concepts discussed in session 3. Allow participants to share their personal experience in applying the skills learned from session 3.
  ○ Introduce the fourth skill-building exercise using the finding meaning lesson plan (Appendix H).^15–17^ Use the exercises for energy balance (Appendix I) and gratitude letters (Appendix J) to facilitate skill-building exercises for finding meaning in clerkship work.
  ○ At the conclusion of this session, provide time to summarize the skills learned throughout the workshop series.
  ○ Distribute workshop postsurvey evaluations (Appendix K) to participants. Feedback can be used to restructure future workshops. In addition, one may choose to redistribute the CD-RISC (Appendix A) and have participants analyze their scores at the conclusion of the series compared to their scores at the initiation of the series (optional).^19^
• Social media reflection.
  ○ Establish a secure microblogging platform for the discussion group (e.g., Yammer or Canvas).
  ○ Create a private group and invite students.^6,7^
  ○ Provide instructions to the students, using the Social Media Positive Psychology Reflection instructions (Appendix L).
  ○ Start the group with a moderator post.
  ○ Follow up students' posts with moderator comments.

## Results

This workshop series has been delivered in its full content during the 2017–2018 academic year at University A with 74 clerkship students (curriculum survey response rate: 72%, 53 out of 74) and at University B with 70 clerkship students (curriculum survey response rate: 84%, 59 out of 70). It is underway during the 2018–2019 academic year at both institutions. Anonymous curriculum evaluation surveys were distributed to all medical students completing the workshop sessions. Focus groups were also conducted with approximately seven students at University A.

At University A, there was an increase in students' CD-RISC score following the workshop series (preworkshop *M* = 26.9, *SD* = 6.5, vs. postworkshop *M* = 29.6, *SD* = 5.3; *p* = .019). More than half of students agreed or strongly agreed that the sessions had been an open forum for reflection (55%, 29 out of 53) and allowed them to connect with peers (51%, 27 out of 53). Fifty-one percent (27 out of 53) thought having time to discuss these topics was valuable. Overall, 63 students posted to the microblogging positive psychology activity (85% response rate, 63 out of 74). Less than half (41%, 25 out of 61) the students thought the microblogging reflections should continue, and only 5% enjoyed using microblogging for reflection. Themes in students' written comments and focus groups included connecting with peers and feeling less alone. Some students commented that holding sessions with clerkship directors present was not ideal for them, whereas others felt they had access to other outlets for these discussions. Many students appreciated that attention was paid to their wellness during clerkships. Students enjoyed reading what others posted on the microblogging reflection but reported anxiety and concern about writing their own posts given the lack of anonymity and self-congratulatory perception.

At University B, there was no significant change in mean CD-RISC resilience scores between the pre- and postintervention students. The majority of students agreed or strongly agreed that the sessions provided an open forum for reflection (77%, 54 out of 70) and allowed them to connect with their peers (65%, 46 out of 70). The majority of students found the sessions to be useful (63%, 44 out of 70), agreed that they should continue the following year (77%, 54 out of 70), and found the sessions to be a valuable use of time (77%, 54 out of 70). The use of Canvas as a blogging platform for reflection was not a mandatory portion of the sessions at University B; however, it was made available for reflection and discussion outside of the workshop sessions. No students took advantage of the platform. Students' comments overwhelmingly viewed the sessions as a time to reflect and share experiences with their peers and valued a more unstructured workshop that maximized peer discussions. An additional theme in students' feedback was that end-of-day sessions decreased overall satisfaction and that scheduling sessions during normal clerkship didactics made sessions appear mandatory, despite stating their voluntary nature. In addition, several students commented that having sessions during clerkship time took away from opportunities for studying or self-directed wellness pursuits.

## Discussion

This medical student resilience curriculum during clerkship training, composed of small-group skill-building sessions, was well received, was perceived as valuable, and provided students with additional support systems that may not have been obvious at the start of their clerkship training. It addressed a growing concern with burnout in medical students and provided a practical approach to helping students learn resilience skills to manage stress at the time of exposure to stressful clinical training. Furthermore, the presurvey for the curriculum allowed for student-driven selection of content areas given its focus on student-perceived stressors during clinical training. This survey and these sessions could be adapted for delivery in a variety of core clerkships, as well as across the medical education continuum.

This curriculum targeted clinical stressors highlighted by medical students as a springboard to teach resilience skills. The curriculum was valuable for half of students, and it is possible that resilience was improved at one academic center. However, resilience scores did not improve at the second academic center, and further studies are needed to fully assess the impact of skills-based training on student resilience. Furthermore, resilience and burnout have been correlated in studies conducted in graduate medical education, but not specifically in clerkship students. While demographic data, burnout scores, and resilience scores are collected longitudinally at both institutions involved in these studies, these data were not specifically used to track learners within our educational intervention. Future research should be aimed at tracking burnout and resilience scores for learners participating in and opting out of wellness and resilience-based curricula to fully assess the impact of these educational interventions.

There were differences between outcomes at the two educational sites. Student satisfaction with the curriculum at University A was slightly lower, but there was a higher overall participation in the social media reflection. At University B, there was higher satisfaction but no participation in the social media reflection. There are several possible reasons for these differences. At University A, the clerkship director was directly involved in delivering the small-group sessions, which may have changed student perception of the sessions being an open forum for discussion. At University B, all sessions were delivered by faculty involved in wellness activities for the students but not directly involved in any formal assessment. This may have allowed for more open reflection during small-group discussions. The social media platforms differed between the universities. University B used the Canvas platform, which stored students' formal curricular content but was not routinely used for blogging or student communication. Given the voluntary nature of the social media reflection, the use of a social media platform that was unfamiliar to the students may have limited their participation at University B. Students at University A used the Yammer platform, which was familiar to them prior to the development of the curriculum and may have led to higher rates of participation. Future studies should look at students' preferences regarding facilitators for sensitive workshop sessions, as well as their preferred platform for social media reflection.

A challenge for both universities was constructing a setting conducive to comfortable reflection for all learners. Not all students found these sessions necessary. While students appreciated educators focusing on their wellness, elective sessions outside of clerkship training or varied sessions for different learners' needs may be more effective. Although students enjoyed reading their colleagues' online reflections, they would have preferred anonymity. This is not surprising as Anderson and colleagues also found concerns around microblogging and sense of a safe learning environment in senior medical students.^21^

A strength of this curriculum is its focus on practical skill building to help mitigate distress given that the curriculum occurs during clerkship training. In addition, this workshop series allows students to create a shared experience and fosters an environment of peer support within clerkship training, which is often viewed as a more isolating time during medical school. The curriculum can be implemented using standard clerkship infrastructure (teaching time, space) and does not require additional funding. Since the sessions are highly interactive, they can adapt to fit any stage of medical school training. However, faculty facilitators should expect to prepare in advance for these workshops, have at minimum a familiarity with positive psychology or mindfulness techniques, and be removed from the grading process for clerkship students to create an ideal learning environment. Furthermore, we did not specifically prepare learners for the content of each session in advance. This is a weakness of our curriculum, and future adaptations include an email to clerkship students regarding the content of upcoming workshops. This would require additional time from faculty facilitators if implemented at their own institution.

This workshop series does not allow trainees to demonstrate competency in any of the skill-building exercises. This is a limitation of our study to date, as it is still unclear to what extent scores on the CD-RISC fluctuate based on time of year, rotation, and outside educational stressors. This was the only objective measure of resilience collected during our curriculum. Further research must focus on identifying ways to quantify utilization of resilience skills in clinical encounters and the overall impact of adopting these skills on future resilience and burnout scores. The participation of our learners was voluntary, and any utilization of these skills outside of the workshop setting was not assessed. In addition, these workshops allow identification of areas where support is needed during clerkship training, but without close communication with clerkship directors and medical school educational leadership, there is not a standard process for quality improvement within these workshops.

A limitation of this curriculum is its use as a brief, time-limited intervention that is not longitudinal. More in-depth practice and training may be required to reliably impact levels of resilience. Furthermore, while resilience is a promising area for improving medical student wellness, it is unlikely to mitigate burnout in isolation. A multipronged approach is necessary to address other aspects of the clinical learning environment that impact trainee burnout including nonclinical work, control over clerkship schedule, and access to mental health and stress-relief resources. Future studies should assess the impact of demonstrated mastery in resiliency skills on levels of stress and burnout, as well as evaluate the impact of longitudinal integration of resilience training across the medical education continuum.

## Appendices

Connor-Davidson Resilience Scale Access.docxCurriculum Presurvey.docxExercise - Goals and Expectations.docxLesson Plan - Difficult Team.docxPocket Card - Difficult Team Interactions.docxLesson Plan - Disappointments and Setbacks.docxExercise - Compassionate Listening.docxLesson Plan - Finding Meaning.docxExercise - Energy Balance.docxExercise - Gratitude Letter.docxCurriculum Postsurvey.docxSocial Media - Positive Psych Reflection Instructions.docx
All appendices are peer reviewed as integral parts of the Original Publication.
