# Supplementary material for: A Curriculum to Teach Resilience Skills to Medical Students During Clinical Training
Source: MedEdPORTAL. 2020 Sep 30;16:10975. doi: 10.15766/mep_2374-8265.10975 (PMC7526502; doi:10.15766/mep_2374-8265.10975)
Supplement: Supplementary file 1 — Connor-Davidson Resilience Scale Access.docxCurriculum Presurvey.docxExercise - Goals and Expectations.docxLesson Plan - Difficult Team.docxPocket Card - Difficult Team Interactions.docxLesson Plan - Disappointments and Setbacks.docxExercise - Compassionate Listening.docxLesson Plan - Finding Meaning.docxExercise - Energy Balance.docxExercise - Gratitude Letter.docxCurriculum Postsurvey.docxSocial Media - Positive Psych Reflection Instructions.docx [file mep_2374-8265.10975-s001.zip › L. Social Media - Positive Psych Reflection Instructions.docx]

**Social Media Positive Psychology Reflection Instructions**

Instructions for instructor:

- Create a secure social media group for you and your students using a platform such as, yammer (<https://www.yammer.com>) or canvas. Create a private group and invite students.
- Instruct students to participate
- Start of the group with your own personal shout-out
- Make sure you read the posts and comment on them
- Try to add additional posts to keep the discussion going

Instructions for students:

- Post 1 sentence that is HIPAA free about a positive accomplishment with a patient
  - basically a personal shout-out
- You can repeat what others have said
- You can ask each other questions
- There will be a faculty moderator
- Posts will be deleted at the end of the academic year
- None will be re-cited for any purpose
