# Supplementary material for: A Curriculum to Teach Resilience Skills to Medical Students During Clinical Training
Source: MedEdPORTAL. 2020 Sep 30;16:10975. doi: 10.15766/mep_2374-8265.10975 (PMC7526502; doi:10.15766/mep_2374-8265.10975)
Supplement: Supplementary file 1 — Connor-Davidson Resilience Scale Access.docxCurriculum Presurvey.docxExercise - Goals and Expectations.docxLesson Plan - Difficult Team.docxPocket Card - Difficult Team Interactions.docxLesson Plan - Disappointments and Setbacks.docxExercise - Compassionate Listening.docxLesson Plan - Finding Meaning.docxExercise - Energy Balance.docxExercise - Gratitude Letter.docxCurriculum Postsurvey.docxSocial Media - Positive Psych Reflection Instructions.docx [file mep_2374-8265.10975-s001.zip › B. Curriculum Presurvey.docx]

Resilience Survey

Please do not put your name on this survey; we will be using your anonymous responses for research purposes. Your participation is voluntary.

**How much do you agree with the following statements as they apply to you over THE LAST MONTH (Select one):**

|  | Not True at All | Rarely True | Sometimes True | Often True | True Nearly All the Time |
| --- | --- | --- | --- | --- | --- |
| I am able to adapt when changes occur. |  |  |  |  |  |
| I can deal with whatever comes my way. |  |  |  |  |  |
| I try to see the humorous side of things when I am faced with problems. |  |  |  |  |  |
| Having to cope with stress can make me stronger. |  |  |  |  |  |
| I tend to bounce back after illness, injury, or other hardships. |  |  |  |  |  |
| I believe I can achieve my goals, even if there are obstacles. |  |  |  |  |  |
| Under pressure, I stay focused and think clearly. |  |  |  |  |  |
| I am not easily discouraged by failure. |  |  |  |  |  |
| I think of myself as a strong person when dealing with life’s challenges and difficulties. |  |  |  |  |  |
| I am able to handle unpleasant or painful feelings like sadness, fear and anger. |  |  |  |  |  |

**Which of the following clinical events have you experienced during your clinical time? (Select all that apply)**

|  | Dealing with difficult patient encounters |
| --- | --- |
|  | Codes |
|  | Unanticipated patient deaths |
|  | Difficult family discussions |
|  | System issues |
|  | Poor team dynamics |
|  | Medical errors |
|  | Chronic opioid management |
|  | Difficult encounters with other staff |

**Which are the 3 most stressful clinical events for you? (Select 3 from below):**

|  | Dealing with difficult patient encounters |
| --- | --- |
|  | Codes |
|  | Unanticipated patient deaths |
|  | Difficult family discussions |
|  | System issues |
|  | Poor team dynamics |
|  | Medical errors |
|  | Chronic opioid management |
|  | Difficult encounters with other staff |

|  | Immediately |
| --- | --- |
|  | Later the same day |
|  | In the following days to weeks |
|  | After the rotation is over |
|  | I would prefer not to discuss with my team |

**After difficult clinical events, when would you prefer that your team discuss them? (Select one):**

**After difficult clinical events, how often do you reflect on them individually? (Select one):**

|  | Never |
| --- | --- |
|  | Rarely |
|  | Sometimes |
|  | Often |
|  | Very Often |

**Who have you talked to about difficult clinical events? (Select all that apply):**

|  | Team attending |
| --- | --- |
|  | Team resident |
|  | Team intern |
|  | Ombudsmen |
|  | Medical school dean |
|  | Faculty mentor |
|  | Clerkship Director |
|  | Significant other |
|  | Family |
|  | Fellow student |
|  | Friend outside of medical school |
|  | Other (please list): |

|  |
| --- |

**If you didn’t talk with your team attending, why not? (not required to respond)**

**Please state how strongly you agree or disagree with the following statements (mark one):**

|  | Strongly Disagree | Disagree | Neutral | Agree | Strongly Agree |
| --- | --- | --- | --- | --- | --- |
| I have the skills necessary to personally cope with difficult clinical events (i.e. unexpected deaths, difficult patients, medical errors) |  |  |  |  |  |
| I have the skills necessary to manage stress and prevent burnout |  |  |  |  |  |
| I have the skills necessary to cope with setbacks and failures |  |  |  |  |  |
| Difficult clinical events affect my well being |  |  |  |  |  |
| I feel comfortable talking about stress and burnout with my peers |  |  |  |  |  |
| I feel comfortable talking about medical errors I have been involved in, with my peers |  |  |  |  |  |
| I think resilience training (learning how to adapt well to challenges) would be helpful |  |  |  |  |  |
| I have had sufficient resilience training (learning how to adapt well to challenges) |  |  |  |  |  |

**We are designing workshops to teach students resilience skills, mark the 3 most important topics to you (areas that you need more training in)? (Select ONLY 3)**

|  | Setting realistic goals |
| --- | --- |
|  | Delivering bad news |
|  | Dealing with disappointments and setbacks |
|  | Finding meaning in your daily work |
|  | Managing expectations |
|  | Coping with medical errors |
|  | Dealing with loss |
|  | Coping with difficulty patient interactions |
|  | Coping with difficult team interactions (i.e. student, resident, attending) |
|  | Feeling gratitude in daily work |
|  | Other (please list): |

**When do you think this resiliency education would be most beneficial? (select 1)**

|  | Before 3rd Year |
| --- | --- |
|  | During 3rd Year |
|  | During 4th Year |

**Suggestions: (optional)**

|  |
| --- |

**Select one response to below questions**

|  | Male |
| --- | --- |
|  | Female |
|  | Non-binary |
|  | Prefer not to say |

**Sex:**

|  | 18-25 |
| --- | --- |
|  | 26 or older |
|  | Prefer not to say |

**Age:**

|  | Science |
| --- | --- |
|  | Non-Science |

**Undergraduate major:**

|  | MS3 |
| --- | --- |
|  | MS4 |

**Year in School:**

|  | Traditional - straight from undergraduate education |
| --- | --- |
|  | Non-Traditional - took time off between undergraduate education and medical school |

**Path to medical school**:
