# Supplementary material for: A Curriculum to Teach Resilience Skills to Medical Students During Clinical Training
Source: MedEdPORTAL. 2020 Sep 30;16:10975. doi: 10.15766/mep_2374-8265.10975 (PMC7526502; doi:10.15766/mep_2374-8265.10975)
Supplement: Supplementary file 1 — Connor-Davidson Resilience Scale Access.docxCurriculum Presurvey.docxExercise - Goals and Expectations.docxLesson Plan - Difficult Team.docxPocket Card - Difficult Team Interactions.docxLesson Plan - Disappointments and Setbacks.docxExercise - Compassionate Listening.docxLesson Plan - Finding Meaning.docxExercise - Energy Balance.docxExercise - Gratitude Letter.docxCurriculum Postsurvey.docxSocial Media - Positive Psych Reflection Instructions.docx [file mep_2374-8265.10975-s001.zip › C. Exercise - Goals and Expectations.docx]

Setting Goals and Managing Expectations Exercise

1. Write down the first goal that comes to mind:

- - Quickly write down whether you think attaining this goal will be easy or hard
  - Specifically reflect on your expectations for the path to achieving this goal

2. Think about your original goal and expectations

- - Apply the skills learned and apply a resilient way to approach this goal
  - Set expectations slightly harder than reality
    - How will you identify what a typical experience is for achieving your goal?
  - Break your goal into stages
    - What are small steps that need to be achieved in order to meet your goal?
  - Approach easy tasks first
    - Reorder the stages of your goal to focus on east to accomplish tasks first
    - This will help to build early success and motivate for ongoing work towards your goal
