# Supplementary material for: A Curriculum to Teach Resilience Skills to Medical Students During Clinical Training
Source: MedEdPORTAL. 2020 Sep 30;16:10975. doi: 10.15766/mep_2374-8265.10975 (PMC7526502; doi:10.15766/mep_2374-8265.10975)
Supplement: Supplementary file 1 — Connor-Davidson Resilience Scale Access.docxCurriculum Presurvey.docxExercise - Goals and Expectations.docxLesson Plan - Difficult Team.docxPocket Card - Difficult Team Interactions.docxLesson Plan - Disappointments and Setbacks.docxExercise - Compassionate Listening.docxLesson Plan - Finding Meaning.docxExercise - Energy Balance.docxExercise - Gratitude Letter.docxCurriculum Postsurvey.docxSocial Media - Positive Psych Reflection Instructions.docx [file mep_2374-8265.10975-s001.zip › D. Lesson Plan - Difficult Team.docx]

**Difficult Team Dynamics Reflection Session Lesson Plan**

Student Instructions:

In preparation for this session think about a time you’ve faced challenging team dynamics. This could have been during any rotation or even a team unrelated to medicine. Take a few minutes to write about your experience or gather your thoughts. Please avoid using names for anonymity. You may write or think about how you overcame it or responded to it. This is certainly not graded but is a required part of the clerkship.

Hopefully this exercise will help you step back and consider some of the things that we learn from our experiences and often don’t realize. We will also use them to help teach you strategies to help difficult team interactions.

Session Notes/Plan:

1. Intro:

- “Conflict, like trust and interdependence, is also a necessary part of becoming a real team.”^1^
- Conflict can be defined as a disagreement within oneself or between people that causes harm or has the potential to cause harm. Usually due to : emotions, personal history, communication or values.^2^

2. Group Reflection (have someone read scenario or read for the group)

- You have a new senior resident as your resident on the wards. He has been talking about the patient updates only with the intern (not you). He hasn’t been making time to review your presentations in the morning before rounds. When he reviews the H&P’s the night before, he gives you feedback but when the attending disagrees, he doesn’t acknowledge that he had recommended it.
- Questions to discuss as a large group
  - What factors may be at play?
  - How would you approach this?
  - How would you give feedback?

3. Group reflection Debrief

- Discuss strategies for conflict management
  - Compete
  - Collaborate
  - Avoid
  - Accommodate
    - Saltman DB, O’Dea NA, Kidd MR. Conflict Management: a primer for doctors in training. Postgrad Med J. 2006; 82:9-12.
- Give examples of when may be appropriate vs. another (great to use personal examples)
  - Avoid may be okay for very short term relationship i.e. 1 shift overlap
  - Collaborate best for 2 students on same team
- Discuss personal factors in physicians that can lead to conflict
  - Attitudes – burnout, insecurity, uncertainty, negative bias, time pressure
  - Conditions- anxiety/depression, sleep deprivation, overwork, personal health problems, situation stressors
  - Knowledge – limited medical knowledge, limited knowledge about teamwork
  - Skills- difficult with communication, easy frustration, difficulty expressing empathy
    - Lorenzetti RC at al. Managing Difficult Encounters: Understanding Physician, Patient, and Situational Factors. Am Fam Physician. 2013;87(6):419-425.
- Discuss Ende’s Feedback Principles and give examples
  - Work as allies with common goals
  - Well-timed and expected
  - Based on first-hand data
  - Regulated in quantity
  - Limited to behaviors that are remediable
  - Use descriptive non-evaluative language
  - Based on specific examples not generalizations
  - Deal with decisions and actions rather than assumed intentions or interpretations
    - Ende J. Feedback in Clinical Medical Education. JAMA. 1983; 250:777-81
- Review INTERVENE Framework (give out pocket card to participants and refer to it)

| **I** | **Intervene early** | *Early intervention makes it easier* |
| --- | --- | --- |
| **N** | **Note the best management strategy** | *Avoid, accommodate, compete, collaborate* |
| **T** | **Think through other factors at play** | *Don’t take it personally* |
| **E** | **Elect the encounter** | *Choose appropriate setting and time for discussion* |
| **R** | **Regulate the amount of feedback** | *Think about what is most important* |
| **V** | **Verify the common goal** | *See the larger mission for the team* |
| **E** | **Explore intentions** | *Don’t assume intentions* |
| **N** | **Narrate specific examples and provide alternatives** | *Point out specific examples of actions/behavior, personality cannot be changed* |
| **E** | **Enlist help from others** | *When needed get the right help involved* |

3. Partner Exercise

- Have students break off into self-selected pairs or groups of 3
- Students should share their experiences with a difficult team as prompted before the session
- Then discuss how they responded to the situation or tried to solve it and answer the following questions:
  - Which conflict strategy did you use?
  - What other factors could have been at play?
  - What’s another way you could have approached the problem?

4. Partner Exercise Debrief

- Have willing groups share their discussion with the larger group. Point out concepts discussed along the way and ask the rest of the group for ideas or feedback if there is uncertainty.

5. Conclusion

- Ask students for final thoughts or questions
- Remind them of resources for help at your institution: resident, intern, attending, chief resident, clerkship director, dean, ombudsmen, fellow students, career advisor, faculty mentor, supervisor, etc.
- Discuss what the process is when students meet with Deans or clerkship directors with problems. How is it handled? Does the person get notified? Make this transparent.

6. Addressing the System

- Remind students that you are also trying to help improve the system to support their education. Discuss how and that you are committed to this.

References:

1. Katzenbach JR, Smith DK. *The Wisdom of Teams: Creating the High-Performance Organization*. New York, NY: Harper Business; 1993.

2. Harolds J, Wood BP. Conflict Management and Resolution. *J Am Coll Radiol*. 2006; 3:200-206.
