# Supplementary material for: A Curriculum to Teach Resilience Skills to Medical Students During Clinical Training
Source: MedEdPORTAL. 2020 Sep 30;16:10975. doi: 10.15766/mep_2374-8265.10975 (PMC7526502; doi:10.15766/mep_2374-8265.10975)
Supplement: Supplementary file 1 — Connor-Davidson Resilience Scale Access.docxCurriculum Presurvey.docxExercise - Goals and Expectations.docxLesson Plan - Difficult Team.docxPocket Card - Difficult Team Interactions.docxLesson Plan - Disappointments and Setbacks.docxExercise - Compassionate Listening.docxLesson Plan - Finding Meaning.docxExercise - Energy Balance.docxExercise - Gratitude Letter.docxCurriculum Postsurvey.docxSocial Media - Positive Psych Reflection Instructions.docx [file mep_2374-8265.10975-s001.zip › E. Pocket Card - Difficult Team Interactions.docx]

**INTERVENE**

*A framework to help difficult team interactions*

| **I** | **Intervene Early** |
| --- | --- |
| **N** | **Note the Best Management Strategy** |
| **T** | **Think through other factors at play** |
| **E** | **Elect the encounter** |
| **R** | **Regulate the amount of feedback** |
| **V** | **Verify the common goal** |
| **E** | **Explore intentions** |
| **N** | **Narrate specific examples and provide alternatives** |
| **E** | **Enlist help from others** |

Bird, Tomescu & Pincavage, 2018 Ende J. JAMA. 1983; 250: 777-81.

**INTERVENE**

*A framework to help difficult team interactions*

| **I- Intervene early** | *Early intervention makes it easier* |
| --- | --- |
| **N-Note strategy** | *Avoid, accommodate, compete, collaborate* |
| **T- Think through factors** | *Don’t take it personally* |
| **E- Elect the encounter** | *Choose appropriate setting and time for discussion* |
| **R- Regulate feedback** | *Think about what is most important* |
| **V- verify goal** | *See the larger mission for the team* |
| **E- Explore intentions** | *Don’t assume intentions* |
| **N – Narrate examples** | *Point out specific examples of actions/behavior, personality cannot be changed* |
| **E- Enlist help** | *When needed get the right help involved* |

Bird, Tomescu & Pincavage, 2018 Ende J. JAMA. 1983; 250: 777-81.
