# Supplementary material for: A Curriculum to Teach Resilience Skills to Medical Students During Clinical Training
Source: MedEdPORTAL. 2020 Sep 30;16:10975. doi: 10.15766/mep_2374-8265.10975 (PMC7526502; doi:10.15766/mep_2374-8265.10975)
Supplement: Supplementary file 1 — Connor-Davidson Resilience Scale Access.docxCurriculum Presurvey.docxExercise - Goals and Expectations.docxLesson Plan - Difficult Team.docxPocket Card - Difficult Team Interactions.docxLesson Plan - Disappointments and Setbacks.docxExercise - Compassionate Listening.docxLesson Plan - Finding Meaning.docxExercise - Energy Balance.docxExercise - Gratitude Letter.docxCurriculum Postsurvey.docxSocial Media - Positive Psych Reflection Instructions.docx [file mep_2374-8265.10975-s001.zip › F. Lesson Plan - Disappointments and Setbacks.docx]

**Dealing with Disappointments & Setbacks Session**

Negative Thinking has been historically viewed as “bad” and positive thinking as “good” but this is only one way of viewing our thoughts. Another way to consider our thoughts is as “teachers” that reveal something different about who we are and how navigate our lives. Learning how to view our thoughts in a more compassionate manner can be a useful skill as we care for our patients, work in challenging systems and manage difficult interactions, disappointments and setbacks. “Compassionate listening”, very different from the typical listening physicians employ, has only one purpose, to allow for the storyteller to unload their heart and suffering. In this type of listening, the physician is not thinking of solutions and ideas or interrupting to share those ideas but is simply listening, with full attention. This type of listening can otherwise be described as paying 100% attention to the person in front of us and often relieves the storyteller of their suffering. “Compassionate listening” cultivates further self-compassion which is essential when dealing with disappointments and setbacks.

Activities:

1. You will be given a journal to practice “thought journaling” where you can write down your thoughts, as you are able, throughout the day.

2. Write a reflection after the 1^st^ and 2^nd^ week of this rotation about any professional or personal interaction that was disappointing with the focus on the following:

- - “What did this moment teach me?”
  - “What do I know now about myself that I didn’t prior to this disappointment/setback?”
  - “What was the most challenging part of this moment?”
  - “How might it inform my work as a future physician and or member of this community?”
  - “How could I view this difficult moment in a different way?”

3. On the last day of the clerkship, we will have a Dealing with Disappointments and Setbacks session where we will discuss the meaning of compassion, review what it means to listen compassionately, you will share one of your reflections with a colleague and also practice “compassionate listening”. Please choose a reflection piece that you feel most comfortable sharing.

**Session Discussion questions after the students have reflected in pairs:**

- - How did it go?
  - What was more challenging, being the listener or story teller?
  - What aspects of compassionate listening were most challenging or easier and why?
  - What aspects of the story telling were most challenging or easier and why?
  - Did anything surprise you about this activity?
  - How do you think this type of journaling/listening and/or story telling will assist you in dealing with a disappointment/setback?

Prior to ending the session engage the students in sharing some of the above reflection questions with the larger group depending on time and student engagement.
