# Supplementary material for: A Curriculum to Teach Resilience Skills to Medical Students During Clinical Training
Source: MedEdPORTAL. 2020 Sep 30;16:10975. doi: 10.15766/mep_2374-8265.10975 (PMC7526502; doi:10.15766/mep_2374-8265.10975)
Supplement: Supplementary file 1 — Connor-Davidson Resilience Scale Access.docxCurriculum Presurvey.docxExercise - Goals and Expectations.docxLesson Plan - Difficult Team.docxPocket Card - Difficult Team Interactions.docxLesson Plan - Disappointments and Setbacks.docxExercise - Compassionate Listening.docxLesson Plan - Finding Meaning.docxExercise - Energy Balance.docxExercise - Gratitude Letter.docxCurriculum Postsurvey.docxSocial Media - Positive Psych Reflection Instructions.docx [file mep_2374-8265.10975-s001.zip › G. Exercise - Compassionate Listening.docx]

Compassionate Listening Exercise

*What is Compassionate Listening?*

It is deep listening which is the kind of listening that can help relieve the suffering of another person. You can call it compassionate listening. You listen with only one purpose: to help the person sharing unload their suffering or “empty their heart”.

Steps:

1. Remember to take a deep breath and remind yourself that this is an opportunity to be completely present with the person before you.

2. As you listen, your mind may start to wander to thoughts you want say to soothe or relieve or solve their problem. When this happens, just gently bring yourself back to the person’s voice, their facial expressions or even how they are sitting in the chair in front of you.

3. Try to look into the person’s eyes as they are speaking and acknowledge you are hearing their story by nodding, leaning in, using appropriate facial expressions for each part of the story, i.e. smile when they are saying something meant to be funny.

4. Try to repeat parts of the story to yourself to fully appreciate the details of the story.

5. Once the person ends their story, all you have to do is thank them for sharing and say you are sorry they are dealing or dealt with something so challenging.

6. Please end by saying you are here for them if they ever needed someone to listen.

Reflection exercise:

- How did this make you feel?
- How did the other person respond?
