# Supplementary material for: A Curriculum to Teach Resilience Skills to Medical Students During Clinical Training
Source: MedEdPORTAL. 2020 Sep 30;16:10975. doi: 10.15766/mep_2374-8265.10975 (PMC7526502; doi:10.15766/mep_2374-8265.10975)
Supplement: Supplementary file 1 — Connor-Davidson Resilience Scale Access.docxCurriculum Presurvey.docxExercise - Goals and Expectations.docxLesson Plan - Difficult Team.docxPocket Card - Difficult Team Interactions.docxLesson Plan - Disappointments and Setbacks.docxExercise - Compassionate Listening.docxLesson Plan - Finding Meaning.docxExercise - Energy Balance.docxExercise - Gratitude Letter.docxCurriculum Postsurvey.docxSocial Media - Positive Psych Reflection Instructions.docx [file mep_2374-8265.10975-s001.zip › H. Lesson Plan - Finding Meaning.docx]

**Reflection Exercise:**

1. **Challenging patient reflection exercise**

**-Discuss personal experience**

**-Preceptor:** normalize that you may be feeling “bad” (stressed; uncomfortable) right now. These are all very strong emotions you released on this piece of paper. Most of these matters were not in your conscious awareness; doing a writing reflection like this brings them into consciousness, which at first can be quite unpleasant. Make no mistake though, these issues, when subconscious, will still manifest, but in ways that is often beyond your control. What I want to normalize is that this first step of awareness is tough because it can trigger your body’s stress response system- your sympathetic nervous system and the cascade of adrenaline and cortisol. Does anyone feel this right now?

1. **Skills for draining experiences**
2. Finding Meaning: Reframing the hard times (challenges) you faced as lessons allows you to look past the “negative” experience itself to how it transformed you- and learn from it.
   1. Can you try not to judge draining experiences as “negative”?
   2. Everything is a lesson necessary to your learning.
   3. **Stonecutter Parable:** 3 stonecutters were working side by side. A young man asked each of them the same question: “What are you doing?” The 1^st^ answered: “Are you blind? I am carving this brick out of this large stone.” The 2^nd^ answered: “I am carving this brick out of this large stone so it can be used to build that beautiful historic church. I’m part of its history.” The 3^rd^ answered: “I am carving this brick out of this large stone so it can be used to build that beautiful church in which many people will worship and connect to their higher guidance.”
3. Some of the draining things in your life & school are out of your control.
   1. These are going to drain you no matter what
   2. BUT, they are especially dangerous because they drain you AND can generate such strong reaction on your part that if you get stuck on them, can drain you further.
   3. Your reaction is all you can control
   4. Fine line: you need to experience the emotion (later) but not perseverate on it.
   5. BOTTOM LINE: Don’t waste energy on trying to control what is not yours to control
4. Brutal honesty time: How much are you draining yourself??? How are you draining yourself? Let’s discuss this because this is something that all high achieving people (especially in our profession) do.
   1. The inner dialogue of a perfectionist can be brutally critical, harsh and destructive
   2. Inner Critic vs Inner Guide
   3. Double edged sword: allows you to set high goals and achieve them, BUT can drain you in times when you need all the support you can get- most of all from yourself
      1. If this is you: Begin to pay attention and discern your Inner Critic
      2. Have the intention to be kinder to yourself.

**Sustaining Experiences in your life outside of school**.

- people
- communities
- Selfish me time:
- hobbies/passions/interests/creative outlets
- How do you reward yourself?
- spiritual/religious/mindfulness practices?
- Brutal honesty time: What are your “non-so-healthy” ways that help you get through your days? but that cause un-healthy consequences and may prevent pro-active coping. The key is to be aware of them and be honest with yourself.

sustaining in your life with patients and in school

- School
- Peers, mentors, staff…
- patients/families
- yourself
- Finally, write down your Mission and Purpose for your life and career. Why did you choose this caregiving path? Purpose is a key component of grit- longevity, resilience.

***Sustaining Lists Reflection Questions***

1. Were there any surprises on your sustaining lists (either school or in life outside of school)? Things you had forgotten?
   1. The more aware you are of this circumstance, the more you can recognize them, slow yourself down and learn to be as fully present in these moments as possible. HOW???
      1. Thirsty sponge: soak it all in
      2. Body language: sit down; open chest; deep breath in
      3. Try to make time daily for what is most meaningful to you (even 5-10 min counts)
2. Staying connected to your Mission and Purpose
   - 1. Carry around something small and symbolic
     2. Cognitive reminders help
