# Supplementary material for: A Curriculum to Teach Resilience Skills to Medical Students During Clinical Training
Source: MedEdPORTAL. 2020 Sep 30;16:10975. doi: 10.15766/mep_2374-8265.10975 (PMC7526502; doi:10.15766/mep_2374-8265.10975)
Supplement: Supplementary file 1 — Connor-Davidson Resilience Scale Access.docxCurriculum Presurvey.docxExercise - Goals and Expectations.docxLesson Plan - Difficult Team.docxPocket Card - Difficult Team Interactions.docxLesson Plan - Disappointments and Setbacks.docxExercise - Compassionate Listening.docxLesson Plan - Finding Meaning.docxExercise - Energy Balance.docxExercise - Gratitude Letter.docxCurriculum Postsurvey.docxSocial Media - Positive Psych Reflection Instructions.docx [file mep_2374-8265.10975-s001.zip › I. Exercise - Energy Balance.docx]

**Finding Meaning and Energy Balance Exercise**

1. *Write a list of things that* ***sustain*** *you in the following areas:*

| Work | Life |
| --- | --- |
|  |  |
|  |  |
|  |  |

2. *Write a list of things that* ***drain*** *you in the following areas:*

| Work | Life |
| --- | --- |
|  |  |
|  |  |
|  |  |

***3. Reflection Questions***

a. Were there any surprises on your sustaining lists (either school or in life outside of school)? Things you had forgotten?

b. How can you be more aware of these?

(tips: The more aware you are of this circumstance, the more you can recognize them, slow yourself down and learn to be as fully present in these moments as possible. Thirsty sponge: soak it all in, Body language: sit down; open chest; deep breath in, Try to make time daily for what is most meaningful to you )

c. How can you stay connected to your Mission and Purpose?

(tips: Carry around something small and symbolic, Cognitive reminders help)
