# Supplementary material for: A Curriculum to Teach Resilience Skills to Medical Students During Clinical Training
Source: MedEdPORTAL. 2020 Sep 30;16:10975. doi: 10.15766/mep_2374-8265.10975 (PMC7526502; doi:10.15766/mep_2374-8265.10975)
Supplement: Supplementary file 1 — Connor-Davidson Resilience Scale Access.docxCurriculum Presurvey.docxExercise - Goals and Expectations.docxLesson Plan - Difficult Team.docxPocket Card - Difficult Team Interactions.docxLesson Plan - Disappointments and Setbacks.docxExercise - Compassionate Listening.docxLesson Plan - Finding Meaning.docxExercise - Energy Balance.docxExercise - Gratitude Letter.docxCurriculum Postsurvey.docxSocial Media - Positive Psych Reflection Instructions.docx [file mep_2374-8265.10975-s001.zip › J. Exercise - Gratitude Letter.docx]

Gratitude letter exercise

*Write a letter to express your gratitude and appreciate someone from your gratitude list*
