# Supplementary material for: A Curriculum to Teach Resilience Skills to Medical Students During Clinical Training
Source: MedEdPORTAL. 2020 Sep 30;16:10975. doi: 10.15766/mep_2374-8265.10975 (PMC7526502; doi:10.15766/mep_2374-8265.10975)
Supplement: Supplementary file 1 — Connor-Davidson Resilience Scale Access.docxCurriculum Presurvey.docxExercise - Goals and Expectations.docxLesson Plan - Difficult Team.docxPocket Card - Difficult Team Interactions.docxLesson Plan - Disappointments and Setbacks.docxExercise - Compassionate Listening.docxLesson Plan - Finding Meaning.docxExercise - Energy Balance.docxExercise - Gratitude Letter.docxCurriculum Postsurvey.docxSocial Media - Positive Psych Reflection Instructions.docx [file mep_2374-8265.10975-s001.zip › K. Curriculum Postsurvey.docx]

We recently piloted reflection sessions during the clinical clerkships. These questions are regarding that curriculum:

1. How much do you agree with the following about these sessions? (mark one)

|  | Strongly disagree | Disagree | Neutral | Agree | Strongly agree | N/A |
| --- | --- | --- | --- | --- | --- | --- |
| These sessions should be continued |  |  |  |  |  |  |
| I found these sessions useful |  |  |  |  |  |  |
| I integrated something I learned from these sessions |  |  |  |  |  |  |
| These sessions provided an open forum for reflection |  |  |  |  |  |  |
| These sessions allowed me to connect with my peers |  |  |  |  |  |  |
| Having time to discuss these topics was valuable |  |  |  |  |  |  |
| These sessions helped me feel I’m not alone |  |  |  |  |  |  |
| These sessions helped me feel more comfortable discussing my experiences with peers |  |  |  |  |  |  |
| I thought the number of sessions was sufficient for my clerkship year |  |  |  |  |  |  |

1. Did you post to the reflection discussion on *** during the medicine clerkship? ___ yes ___ no
2. Did you read the reflection discussion on *** during the medicine clerkship? ___ yes ___ no
3. How much do you agree with the following about the reflection discussion on ***? (mark one)

|  | Strongly disagree | Disagree | Neutral | Agree | Strongly agree | N/A |
| --- | --- | --- | --- | --- | --- | --- |
| These discussions should be continued |  |  |  |  |  |  |
| I found the discussion helpful |  |  |  |  |  |  |
| I enjoyed sharing experiences with peers |  |  |  |  |  |  |
| I enjoyed using social media for reflection |  |  |  |  |  |  |
| These discussions took too much time |  |  |  |  |  |  |

1. What did you like most about these sessions and/or the online discussion group?
2. What parts of the sessions did you not like? Why?
3. What should be changed?
4. Other comments/ideas?
